# Lateral facial thread lifting procedure with temporal anchoring in deep temporal fascia: Anatomical perspectives of superficial temporal artery

**DOI:** 10.1111/srt.13587

**Published:** 2024-01-26

**Authors:** Kyu‐Ho Yi, Seung‐Min Oh

**Affiliations:** ^1^ Division in Anatomy and Developmental Biology Department of Oral Biology Human Identification Research Institute BK21 PLUS Project Yonsei University College of Dentistry Seoul Republic of Korea; ^2^ Maylin Clinic (Apgujeong) Seoul South Korea; ^3^ Gangnam ON clinic Seoul South Korea

**Keywords:** cog thread, deep temporal fascia, parietal branch, superficial temporal artery, temporal anchoring, temporal branch, thread lifting

## Abstract

**Introduction:**

Thread lifting is a non‐surgical cosmetic procedure that utilizes threads to lift and tighten sagging skin on the face. In Lateral face lifting with anchoring technique, the threads are inserted into the skin and anchored in place to provide support to the skin at artery free zone. This technique utilizes a long cog thread, allowing for stronger fixation points. The optimal location for thread anchoring is in the fascia of the treatment area.

**Method and materials:**

The study was performed with twelve cadavers with twenty‐four specimens of head from cadavers and was processed using phosphotungstic acid‐based contrast enhancement micro‐computed tomography and conventional computed tomography. The superficial temporal artery with branches of parietal and temporal were then observed with image Slicer program to analyze the safe anchoring place for the deep temporal fascia. The main branch was selected with diameter over 0.3 mm and less than 0.3 mm was regarded as arteriole. Additionally, a case of deep temporal tagging with the Secret Miracle (Hyundae Meditech Co., Ltd., South Korea) has been used for lifting procedures.

**Result:**

The main branch of the parietal branch located posteriorly was located mean of ‐13 mm (range of +5.5 mm to ‐23 mm). And the temporal artery ran most anteriorly had mean of 44 mm anteriorly (range of 32 to 59 mm). The safe area for the tagging is at the deep temporal fascia between the superior temporal line and inferior temporal line. The safe range of deep temporal fascia is a vertical line crossing tragus from 1 to 3 cm anteriorly.

**Conclusion:**

By analyzing the result of the superficial temporal artery of parietal and temporal branches the ideal tagging place for the thread anchoring area has been suggested.

## 1 INTRODUCTION

**FIGURE 1:**
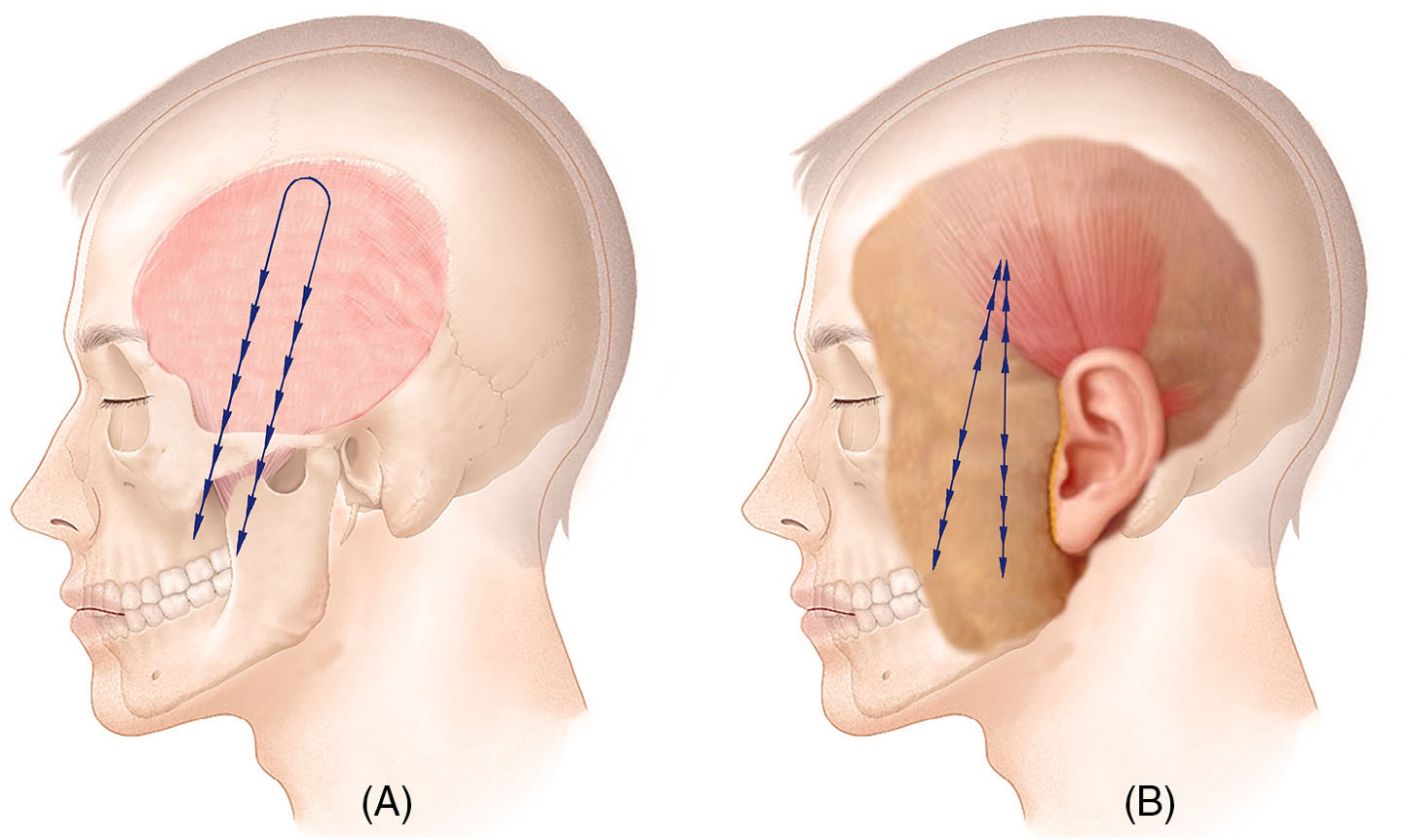
There are two ways to make a fixing point: a method of fixing to the subcutaneous tissue or shallow fascia using a cog (A), and a method of fixing the middle part of a thread without a cog using a long thread (B).

As individuals age, skin laxity and volume loss in the facial region is a prevalent concern. The loss of collagen and elasticity in the skin leads to sagging and drooping.^1^ Non‐invasive cosmetic procedures, such as thread lifting, dermal fillers and botulinum neurotoxin injections, have been proven to effectively address these issues.^2^, ^3^, ^4^, ^5^, ^6^ Thread lifting, in particular, utilizes threads composed of materials such as polydioxanone (PDO), poly‐L‐lactic acid (PLLA) and etc. to lift and tighten sagging skin on the face.^7^ The threads are inserted via a cannula and then pulled taut to lift and smooth wrinkles and sagging skin. Lifting threads utilizes mostly the cog thread characterized by a series of small cogs which are designed to provide improved resistance allowing for stronger fixation points. There are two ways to make a fixing point: a method of fixing the subcutaneous tissue or shallow fascia using a cog, and a method of fixing the middle part of a thread without a cog using a long thread (Figure 1). The one side of the cog usually works as fixation point or anchoring point. The optimal location for thread anchoring is in the deep temporal fascia.^8^ Anchoring the thread in the subcutaneous fat layer or superficial temporal fascia results in a weaker fixation force.^8^ However, by anchoring the thread in the deep temporal fascia, which is the most taut fascia in the temporal region, the procedure is more effective and efficient.

Threadlifting procedures that target the deep temporal fascia are considered optimal, however, it is important to note that there is a risk of damaging the superficial temporal artery, which is a vital anatomical structure that must be avoided.^9^, ^10^, ^11^ Temporal artery damage can be avoided if the skin is incised through a combination of surgical methods and anchoring is performed while directly looking at it. However, since most thread lifting uses a blind technique, temporal artery damage may occur. Despite the importance of this anatomical structure, there is limited research on the branching of the superficial temporal artery regarding thread lifting procedures. It is essential for practitioners to be aware of safe anchoring points for thread procedures targeting the deep temporal fascia to avoid any potential damage to the superficial temporal artery.

## 2 METHODS

Written informed consent for the use of the cadaver and related materials for the purpose of research was provided by all donors or authorized representatives. The authors sincerely thank those who donated their bodies to science so that anatomical research could be performed. Results from such research can potentially increase mankind's overall knowledge that can then improve patient care. Therefore, these donors and their families deserve our highest gratitude.

**FIGURE 2:**
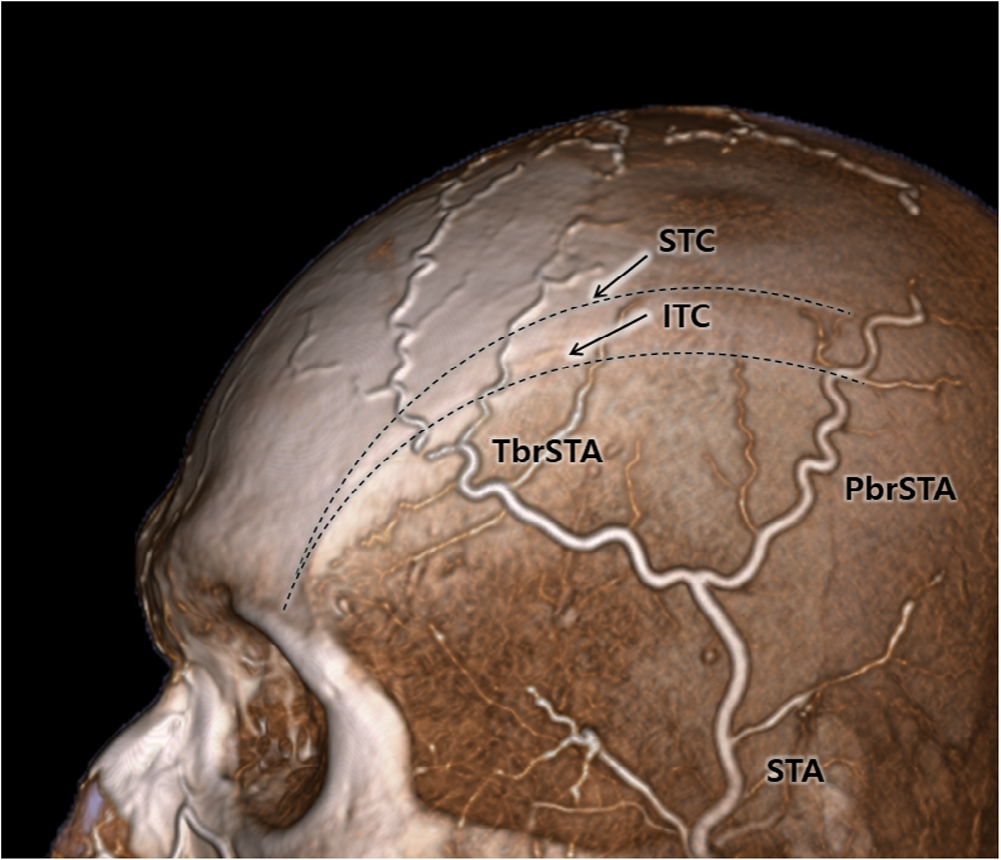
Computed tomography angiography three‐dimensional reconstruction showing the superficial temporal artery (STA) with parietal (PbrSTA) and frontal branches (TbrSTA). The superior temporal crest (STC) and inferior temporal crest (ITC) are guided as dotted line.

Twelve embalmed cadavers (5 males and 7 females; mean age, 73.4 years) were dissected to obtain superficial temporal artery specimens for micro‐CT imaging (Figure 2). The cadavers had no trauma, surgery, or deformity of the head region. Dissection was conducted by carefully removing the skin, subcutaneous tissue, deep fascia, muscles, and superficial vessels. The superficial temporal arterial structures were exposed to examine the possible anatomical structures after which PTA staining was performed.

Furthermore, the patient, a 58‐year‐old woman, sought treatment primarily for facial fat sagging. In this case, a deep temporal tagging procedure was performed using the Secret Miracle (Hyundae Meditech Co., Ltd., South Korea) for the purpose of lifting.

### 2.1 PTA staining

Obtained head specimens fixed by treating with 10% normal buffered formalin for 4 days, followed by dehydration in with increasing concentrations of ethanol from 30% to 70% over the subsequent 3 days. Then the tissues were stained with 1% PTA solution in 70% ethanol and placed on a shaker at 60 rpm for 10 days. The PTA solution used to submerge the harvested head specimens was one part of iodine and two parts of potassium iodide in an aqueous solution. To obtain a concentration of 3%, 1% iodine and 2% potassium iodide were dissolved in double‐distilled water. All instances of PTA staining were performed in 70% ethanol and mixed according to the ratio described above.

### 2.2 Micro‐computed tomography

The superficial temporal artery with branches of parietal and temporal was then observed. The number of the main branch was counted and selected with a diameter over 0.3 mm and less than 0.3 mm was regarded as arteriole.^12^ The measurement was based on the vertical line crossing the tragus. The measurement was taken at the most posteriorly located frontal branch and most anteriorly located parietal branch.

The data was obtained using the Nano & Microfocus X‐ray computed tomography system V|tome|x M (BakerHughes) with minimum detectability of 0.2 μm and a minimum voxel resolution of 0.5 μm, highest power of 300 kV/500 W, and image resolution of image pixel grid 4048 × 4048. The three‐dimensional (3D) reconstructed model was analyzed using a software program (v.5.1.0, Slicer Community, www.slicer.org).

## 3 RESULTS

Superficial temporal arterial structures of the head were observed in every specimen. Detailed descriptions of the superficial temporal arterial structures observed through PTA‐stained micro‐CT are as follows.

The superficial temporal artery has been branched out from the external carotid above the level of the angle of the mandible and runs upward and laterally along the temple. The artery then divides into two branches, the frontal branch and the parietal branch.

The parietal branch of the superficial temporal artery is a branch of the superficial temporal artery that supplies blood to the scalp in the parietal region of the head. It arises from the superficial temporal artery after it has divided from the frontal branch. The parietal branch runs superiorly, paralleling the hairline, and then divides into smaller branches that supply blood to the scalp in the parietal region. These smaller branches can further divide into even smaller branches known as “arterioles” which will nourish the hair follicles and the skin. The parietal branch of the superficial temporal artery is located near the surface of the skin and can be easily palpated. It is also a useful artery for pulse examination.

### 3.1 Parietal branch of superficial temporal artery

**FIGURE 3:**
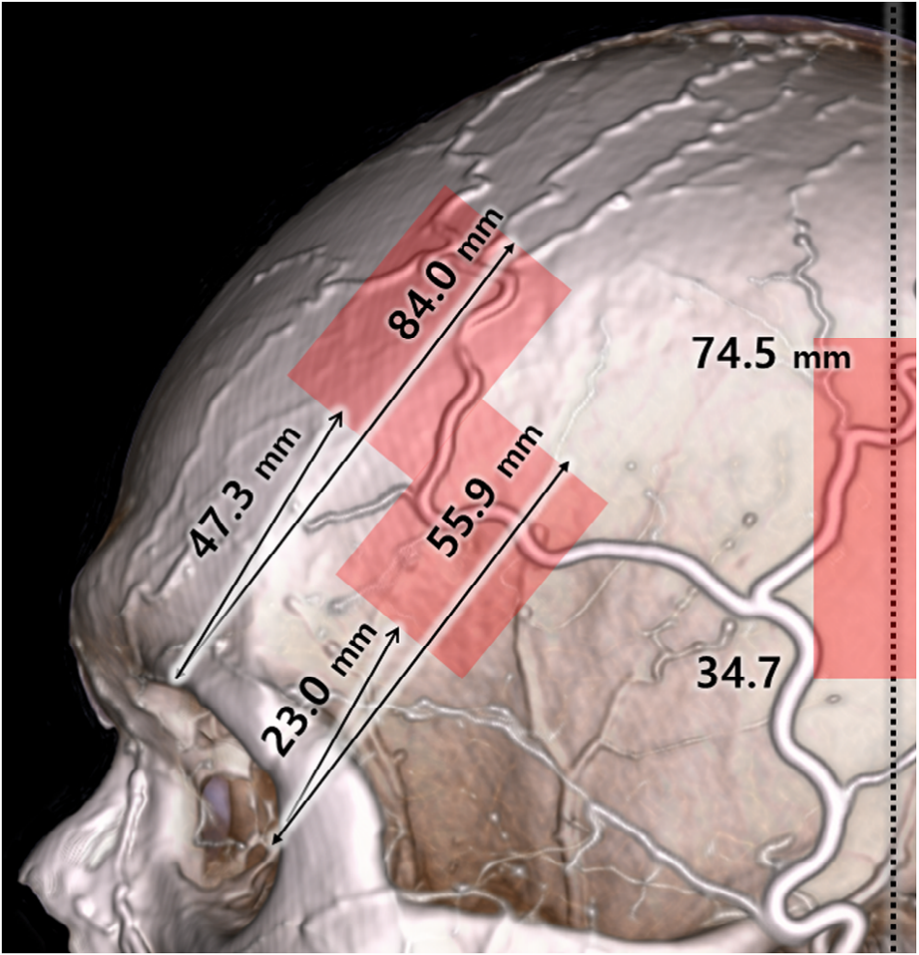
The superficial temporal artery running between the arterial zone are shown with red shaded boxes.

The parietal branch had three to one main branches running superiorly and posteriorly. The parietal branch had two in 18 cases, one in 3 cases and three in 3 cases. All 24 specimens with main branch of the parietal and temporal branches were measured in reference to the vertical line crossing the tragus. The main parietal branch was located mean of ‐13 mm (range of +5.5 mm to ‐23 mm) (Figure 3).

### 3.2 Temporal branch of superficial temporal artery

**FIGURE 4:**
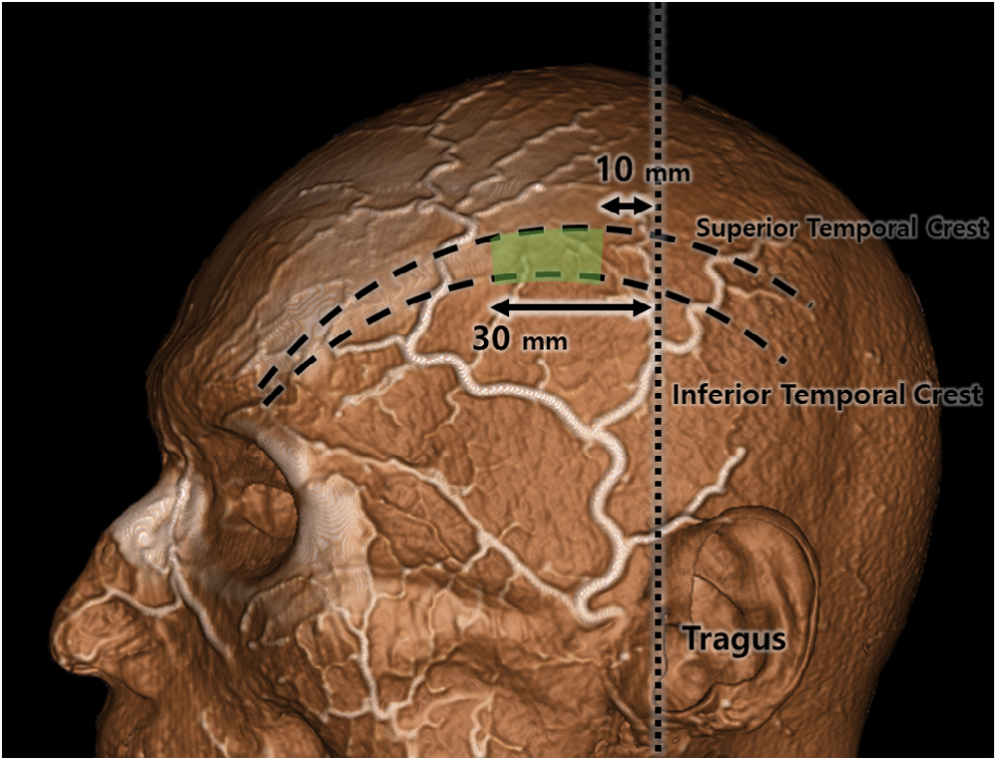
The safe area for the tagging is at the deep temporal fascia at between the superior temporal line and inferior temporal line. The safe range of deep temporal fascia is vertical line crossing tragus from 1 to 3 cm anteriorly. The ideal location of the anchoring region are shaded in green.

The temporal branch of superficial temporal artery had four to two main branches running superiorly and anteriorly. The temporal branch with four were 2 cases, three were 17 cases, and two were 5 cases. The temporal branch ran mean of + 44 mm (range of 32–59 mm) from the vertical line crossing the tragus (Figure 4).

### 3.3 Case

The patient is a 58‐year‐old woman who presented with a primary concern of facial fat sagging. In order to address this issue, a deep temporal fascia tagging thread‐lifting procedure was performed.

**FIGURE 5:**
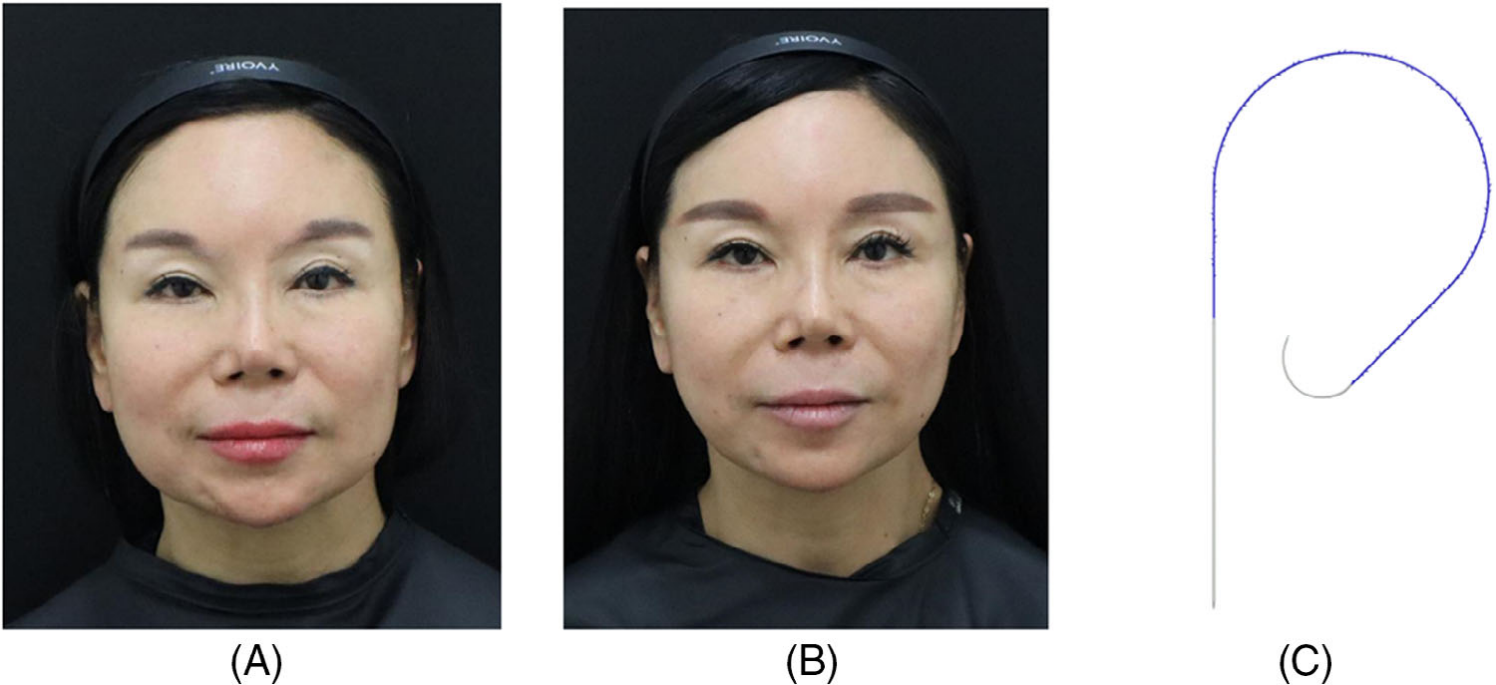
A 58‐year‐old female patient with concerns about facial fat sagging underwent a deep temporal fascia tagging thread lifting procedure. Before (A) and after (B) the thread lifting procedures. The thread used are Secret Miracle (C) from Hyundae Meditech Co., Ltd., South Korea. With two Secret Miracle threads used on each side, and follow‐up imaging was done four weeks post‐surgery.

The Secret Miracle, manufactured by Hyundae Meditech Co., Ltd., South Korea, was utilized for this procedure. This threading material consists of a 140 mm‐long needle with a suture needle on one end and a 320 mm bidirectional cog made of polydioxanone. The side with the suture needle was anchored to the deep temporal fascia, while the long needle was used to thread towards the lower face to perform the lifting procedure. Two Secret Miracle threads were employed on each side for correction. Follow‐up imaging was conducted four weeks after the surgery (Figure 5).

## 4 DISCUSSION

Lifting threads gives lifting of the sagging face and relieving the wrinkles by cog thread anchored at one‐side and dragging the sagging areas.^13^ The optimal anchoring location for thread anchoring is in the deep temporal fascia which taut and located deep to the superficial temporal fascia.^8^

However, it is important to note that there is a risk of damaging the superficial temporal artery, which is a vital anatomical structure that must be avoided. Many previous research had studied running of superficial temporal artery for many surgical flaps and aesthetical purposes like filler and botulinum neurotoxin injections.

The superficial temporal artery and its branches can be visualized using Doppler ultrasound and ultrasonography‐guided marking of the superficial temporal artery would be the most appropriate practical way to avoid complications.^14^ According to Tansatit et al., three arteries were observed on each side of the forehead: the supratrochlear, the supraorbital, and the superficial temporal arteries.^15^ Under ultrasound monitoring, each target artery and its corresponding anastomosis were studied separately through compressions performed in a sequential and accumulative manner. According to the study, ultrasound can assist in the identification and evaluation of all arteries at risk, thereby helping to avoid the occurrence of vascular complications.

According to the research of Koziej et al., in their study of 874 specimens, it was found that the frontal and parietal branches of the superficial temporal artery were present in almost all cases, with 97.6% and 96.4% respectively.^10^ The point where the superficial temporal artery branches off, known as the bifurcation point, was usually located above the zygomatic arch in 79.1% of cases, but it was also found below or on the arch in some instances. There was also a small percentage of cases where there was no bifurcation present. The research also found that the location of the bifurcation point was closely related to the location of the frontal branch, but not the parietal branch. Additionally, the frontal branch was found to have a larger diameter than the parietal branch, suggesting that it is the main branch of the superficial temporal artery.^10^

Koziej et al. determine the location of the parietal branch based on its proximity to the external auditory canal, but the area where it can be found is quite broad, extending from 10^th^ percentile of 34.7 to 90^th^ percentile 74.5 mm above the canal. For the temporal branch, distance between the center of the supraorbital margin and the frontal branch, measured vertically with 10^th^ percentile of 47.3 mm and 90^th^ percentile of 84.0 mm.^16^ They also found out that the anastomosis between the parietal and frontal branches, which is present in 3.6% of patients. Such an anastomosis is located high on the skull and its presence should be considered when performing procedures in this region much over the superior temporal crest.^10^, ^16^

The idea of having a secure point of attachment, known as a fixing point, is a concept that has been passed down through time.^17^, ^18^ Sulamanidze et al. have stated that thread‐lifting procedures can include both a method of attachment that moves and one that is fixed.^19^ The fixed technique discussed here involves creating a strong fixing point on the temporal fascia area.

To achieve optimal lifting effects, the threads must be pulled in a specific direction and a strong fixing point must be present in the opposite direction of the movement caused by aging and gravity. Each thread lifting technique has its own method of creating a fixing point or functions in a similar way. Fixing a thread in the fascia area is considered to be the strongest technique but it is not easy and can result in longer procedure times and side effects such as bleeding. With proper knowledge and skill, safe and effective thread lifting can be performed while minimizing the possibility of bleeding and creating a strong fixing point.

The method for creating a fixing point in thread lifting varies depending on the type of thread lifting used. Many studies do not fully explain the mechanisms behind these methods, such as how bi‐directional cog thread or mono‐thread techniques result in facial lifting.

Histological changes after PDO thread injection—formation of new fibrous connective tissue, connection with existing fibrous tissues, and tissue contraction—can only be explained indirectly.^20^ However, it is clear that lifting can be achieved if the thread is hung in a relatively firm fascia region, runs in the subcutaneous fat layer, and is then pulled. The ideal method for thread lifting is one that can create a strong fixing point with minimal difficulty and side effects. Recently, the trend has been to use a bi‐directional cog thread to gather tissue in the middle part of the thread and form a point that serves a similar function as a fixing point. This is more accurately described as soft tissue repositioning rather than lifting. Another method is to form a fixing point using the hard ligament tissue in the inner side of the face, by causing the cog thread to be hung in the area of the true ligament.^8^ This special technique can be applied in certain areas and can produce better results than the mono‐thread insertion method.

## 5 CONCLUSION

By analyzing the result of the superficial temporal artery of parietal and temporal branch the ideal tagging place for the thread anchoring area has been suggested. Since, the superficial temporal artery is superficial, meaning it is located near the surface of the skin, and can be easily palpated. It is also a useful artery for pulse examination. The safe area for the tagging is at the deep temporal fascia at between the superior temporal line and inferior temporal line. The safe range of deep temporal fascia is vertical line crossing tragus 1–3 cm from the line.

## CONFLICT OF INTEREST STATEMENT

I acknowledge that I have considered the conflict of interest statement included in the “Author Guidelines.” I hereby certify that, to the best of my knowledge, that no aspect of my current personal or professional situation might reasonably be expected to significantly affect my views on the subject I am presenting.

## ETHICAL APPROVAL

Written informed consent for the use of the cadaver and related materials for the purpose of research was provided by all donors or authorized representatives.

## Data Availability

The data and materials are available upon request to corresponding author.
